# WDR3 undergoes phase separation to mediate the therapeutic mechanism of Nilotinib against osteosarcoma

**DOI:** 10.1186/s13046-025-03456-x

**Published:** 2025-07-11

**Authors:** Minglei Li, Nan Li, Yuying Fan, Zhan Zhang, Long Zhou, Yifan Yu, Man Ni, Mingzi Tan, WanJie Huang, Tong Zhu

**Affiliations:** 1 Department of Pediatric Orthopaedics, Shengjing Hospital of China Medical University, No. 36 Sanhao Street of Heping District, Shenyang, Liaoning, 110004 China; 2 Department of Pediatrics, The Fourth Afflicted Hospital of China Medical University, Shenyang, Liaoning, 110032 China; 3 Department of Pediatrics, Shengjing Hospital of China Medical University, No. 36 Sanhao Street of Heping District, Shenyang, Liaoning, 110004 China; 4 Department of Orthopedics, Shengjing Hospital of China Medical University, Shenyang, Liaoning, 110004 China; 5 Department of General Surgery, Shengjing Hospital of China Medical University, Shenyang, 110004 Liaoning China; 6 College of Veterinary Medicine, Jiangsu Co-innovation Center for Prevention and Control of Important Animal Infectious Diseases and Zoonoses, Yangzhou University, Yangzhou, 225009 Jiangsu China; 7 Department of Gynecology, Liaoning Cancer Hospital & Istitute, No.44 Xiaoheyan Road, Dadong District, Shenyang, Liaoning, 110042 China

**Keywords:** Osteosarcoma, Liquid-liquid phase separation, Prognostic biomarker, WDR3, Nilotinib, IDR mutation

## Abstract

**Background:**

Osteosarcoma is highly invasive with a poor prognosis. The phenomenon of liquid-liquid phase separation (LLPS) can promote the formation of biomolecules and participate in the tumor regulation mechanism. Therefore, mining prognostic markers related to LLPS could allow patients to benefit from targeted therapies.

**Method:**

Microarray analysis was performed to identify LLPS-related biomarkers, followed by the validation of binding interactions between genes and drugs via molecular docking analysis. Functions of key genes were investigated in U2-OS cells and xenograft mice. LLPS of WDR3 were observed by the droplet formation assay and fluorescence recovery after photobleaching. The intrinsically disordered region (IDR) of WDR3 was mutated to disrupt LLPS, which was then rescued by the fusion of hnRNAP1 IDR. Therapeutic mechanism of Nilotinib mediated by LLPS was explored in vitro and in vivo.

**Results:**

Five LLPS-related biomarkers were screened by bioinformatics analyses to predict the osteosarcoma prognosis. These prognostic genes were significantly associated with the immune cell infiltration, tumor immune escape and drug sensitivity. Among them, WDR3 was a prognostic risk factor for osteosarcoma and stably bound to Nilotinib in the molecular docking model. In transfected U2-OS cells and xenograft mice, the downregulation of WDR3 significantly inhibited the malignant progression of osteosarcoma. More importantly, WDR3 could form droplets in U2-OS cells and restore the fluorescence intensity of WDR3 condensates with liquid-like behavior after photobleaching. The mutation in IDR impaired the phase separation ability of WDR3, whereas the fusion with hnRNAP1 IDR rescued the phase separation abnormality caused by WDR3 mutation. Moreover, the treatment with Nilotinib improved the progression of osteosarcoma in vivo and in vitro, while inhibiting the production of WDR3 phase-separated condensates.

**Conclusion:**

WDR3 phase separation involves in the therapeutic mechanism of Nilotinib against osteosarcoma, and thus may serve as a potent biomarker to ameliorate adverse events following osteosarcoma treatment.

**Supplementary Information:**

The online version contains supplementary material available at 10.1186/s13046-025-03456-x.

## Highlights

- LLPS-related prognostic biomarkers including WDR3 can predict the prognosis of osteosarcoma.
- WDR3 and Nilotinib exhibited the stable receptor-ligand binding ability in the molecular docking model.
- WDR3 formed droplets in U2-OS cells and promoted tumor metastasis.
- Nilotinib inhibited osteosarcoma progression by disrupting the phase separation of WDR3.

**Supplementary Information:**

The online version contains supplementary material available at 10.1186/s13046-025-03456-x.

## Introduction

Osteosarcoma is a primary solid bone tumor of mesenchymal origin, predominantly occurs in the metaphyses of skeleton (including the femur and tibia) and knee joints [1–3]. Its incidence exhibits a bimodal distribution, peaking in adolescents and older adults over the age of 60, with 4.4 cases of osteosarcoma diagnosed annually per million people worldwide [4, 5]. The 5-year survival rate for patients with localized tumor can reach 70%, but osteosarcoma is highly aggressive, with distal metastases detected in approximately 15–20% of diagnosed cases, most commonly involving the lungs [6–8]. Unfortunately, the metastatic rate of osteosarcoma exceeds 85% and the 5-year overall survival of metastatic patients is less than 20% [9, 10]. In addition to traditional treatments such as surgery, chemotherapy and radiotherapy, targeted therapy and immunotherapy have also been widely used in the clinical management of osteosarcoma; however, drug resistance remains the major cause of reduced survival time, especially for patients with metastatic phenotype [11, 12]. Genomic instability and aberrations are hallmarks of the majority of osteosarcoma cases [13, 14]; therefore, the development of potential biomarkers from a genetic perspective is an effective strategy to enhance targeted therapies and mitigate drug resistance.

Phase separation is a phenomenon that occurs when a mixture of molecules spontaneously separates into two distinct phases with different compositions and concentrations of specific factors [15, 16]. In cellular physiology, biomolecules aggregate to form droplet-like structures within the intracellular environment through liquid-liquid phase separation (LLPS) [17, 18]. This process relies on the intrinsic properties of proteins (such as specific domains) and the stability of external conditions (such as temperature, pH, and NaCl concentrations) [19]. Typically, the intrinsically disordered region (IDR) drives LLPS through interactions among multiple amino acid residues [20]. This physiological phenomenon creates a specialized microenvironment that regulates various cellular processes, including DNA replication, RNA processing, ribosome biogenesis, apoptosis, and signal transduction, thereby affecting tumor progression [21–23]. It has been reported that the aggregation of core regulatory circuitry and transcriptional machinery proteins on super-enhancers via LLPS can promote the activation of genes associated with osteosarcoma metastasis or drug resistance [24]. The inhibition of MYC-driven super-enhancer signaling can counteract the elimination of the proliferation, migration, and invasion of osteosarcoma cells [25]. Kim et al. demonstrated that ARID1A promotes the oncogenic potential of osteosarcoma through prion-like domain-mediated LLPS [26]. To facilitate the study of LLPS mechanisms, Sun et al. summarized various bioinformatics databases and tools [27], but these approaches have not yet been applied to identify biomarkers in osteosarcoma that may affect disease progression and drug sensitivity.

Therefore, this study employed bioinformatics methods to screen LLPS-related biomarkers with prognostic values for osteosarcoma and explored their associations with the immune landscape and drug sensitivity. Molecular docking was used to simulate binding modalities of prognostic biomarkers and drugs and screen for receptor-ligand complexes with structural stability. Furthermore, the involvement of phase separation of the candidate biomarker in the drug therapy against osteosarcoma was also explored through cell- and animal-based experiments. The biomarkers and their phase separation mechanisms reported in this study are expected to provide novel theoretical insights for the treatment of osteosarcoma.

## Methods

### Bioinformatic analysis

#### Data collection and preprocessing

The GSE16091, GSE21257, and GSE39055 datasets containing survival information and gene expression profiling data from 124 patients with osteosarcoma were collected from the Gene Expression Omnibus (GEO) database, and then merged to remove the batch effect using the ComBat function of sva package in R4.2.2. Furthermore, GSE39058 was screened from GEO as the external validation cohort 1; while 85 samples including survival time and expression matrix data were collected from the TARGET database to serve as the external validation cohort 2. In addition, the GSE126209 dataset containing 12 osteosarcoma samples and 11 normal controls was used to screen for differentially expressed genes (DEGs). Using the limma package in R4.2.2 software, DEGs between osteosarcoma and controls were filtered with thresholds of *P* < 0.05 and|log fold_change| > 1. A total of 3,783 LLPS-related genes were obtained from the DrLLPS (http://llps.biocuckoo.cn/), LLPSDB (http://bio-comp.org.cn/llpsdb/home.html), and PhaSepDB (http://db.phasep.pro/) databases. The intersection of DEGs and LLPS-related gene was visualized by the ggplot2 and VennDiagram packages in R4.2.2.

#### Screening of prognostic biomarkers for constructing a predictive model

Based on the data in the combined dataset, the Cox regression analysis was performed using the coxph function from survival package in R4.2.2 to identify prognosis-related genes. Further application of least absolute shrinkage and selection operator (LASSO) regression using the glmnet package provided the optimal combination of prognosis-related genes with the parameter of lambda.min. To prevent overfitting, 50% of the samples from the combined dataset were used as the internal training set, with the remaining samples serving as the internal validation set. External validation cohorts 1 and 2 were employed to verify the model’s stability. The timeROC and survminer packages were used to plot Kaplan-Meier (KM) and receiver operating characteristic (ROC) curves, respectively, to quantify the model prediction results.

#### Molecular subtype of osteosarcoma

The consensus clustering analysis was performed using ConsensusClusterPlus, and the optimal number of clusters was selected based on the cumulative distribution function (CDF) curves and principal component analysis (PCA) results. The clustering criteria were as follows: the number of samples in each group was relatively consistent; the CDF curves gradually increased; and the samples in each group were aggregated, with obvious differences between groups. Differences in survival status and immune checkpoint expression among subtypes were compared using KM curves and *t* tests.

#### Multidimensional comparison between prognostic risk groups

To predict pathway scores for each sample in the combined dataset, the GSVA package was applied with c2.cp.kegg.v2023.1.Hs.symbols.gmt from the MSigDB database as the background pathway. Subsequently, the limma package was employed to screen for pathways with significant differences in scores between high- and low-risk groups, with a threshold of *P* < 0.05. The expression matrix of immune cell subtypes was also deconvoluted using CIBERSORT to estimate the infiltration abundance of immune cell for each sample in the combined dataset. To discern the likelihood of tumor immune evasion, tumor immune dysfunction and exclusion (TIDE) and microsatellite instability (MSI) scores were calculated using the TIDE online tool. A higher TIDE/MSI score indicates a greater immune evasion potential and a poorer response to immune checkpoint inhibitors. The oncoPredict tool, a drug prediction model developed based on the GDSC database, was used in this study to predict the sensitivity of samples to different drugs. Differences in immune cell infiltration levels, TIDE scores, and drug sensitivities between high- and low-risk groups were assessed using *t* tests, followed by Pearson correlation analyses.

#### Molecular docking

To predict the effective blinding of drug and prognostic biomarkers, molecular docking analysis was accomplished in this study. The 3D structures of the drugs were retrieved from the PubChem database and exported into a PDB format using PyMol software. Following, the charge of the ligand was adjusted and twistable bonds were selected in AutoDockTools-4.2.6 software. For receptors, their gene IDs were retrieved from the Uniprot database. Protein 3D structures were collected from the PDB and AlphaFoldDB databases and imported into PyMol as PDB files, with water molecule sequences removed. Subsequently, a series of processes were performed on the receptor in AutoDockTools-4.2.6, including the removal of the original ligand, addition of hydrogens, optimization of amino acids, and calculation of charges. The active region of the binding pocket for docking was determined based on the original ligand position in the protein receptor, and molecular docking was carried out according to the receptor name, ligand name, coordinates of the docking centroid, and distances derived from AutoDock vina. Valid docking results were output if the binding energy was less than -5.0 kcal/mol and hydrogen bonds between the receptor-ligand complex could be formed.

### Cell culture and treatment

The human fetal osteoblast cell line hFOB (CL-233 h, SAIOS biotechnology) and the osteosarcoma cell line U2-OS (CL-655 h, SAIOS biotechnology) were cultured in DMEM supplemented with 10% fetal bovine serum (16140071, Gibco) and 1% penicillin–streptomycin (C11885500BT, Gibco). Two other osteosarcoma cell lines, HOS (CL-156 h, SAIOS biotechnology) and MG-63 (CL-157 h, SAIOS biotechnology), were cultured in MEM (11095080, Gibco) using the same strategy to validate the expression patterns of five prognostic biomarkers.

To observe the effect of WDR3 silencing on osteosarcoma in vitro, sh-WDR3 was transfected into U2-OS cells by lentivirus using Lipofectamine™ 2000 Transfection Reagent (11668030, Invitrogen). The control and sh-negative control (NC) groups were established as well. The sequences of sh-WDR3 (SS Sequence: GGTTCTCTCTAATCTATAA; AS Sequence: TTATAGATTAGAGAGAACC) were designed using the Designer of Small Interfering RNA website.

To explore the pharmacological mechanism of Nilotinib (SC0209, Beyotime), U2-OS cells were treated with different concentrations of Nilotinib (1µM, 2.5µM, 5µM) for 24 h. Based on the cell viability assay, the optimal concentration of Nilotinib (5µM) was selected to treat the cells for 24 h and examine changes in the cell function and the level of WDR3 phase separation.

### Quantitative real-time polymerase chain reaction (qPCR)

The Trizol (1 mL, 15596018, Invitrogen) was added to cells or tissues to release total RNA. Following the reverse transcription, cDNA was synthesized and used for PCR amplification with specific primers. Sequences of primers are detailed in Supplementary Table 1. On a PCR instrument (CFX Connect, BIO-RAD), the PCR reaction was carried out and underwent 40 cycles (95 ℃, 3 min; 95 ℃, 12 s; 62 ℃, 40 s) of amplification. Relative to glyceraldehyde-3-phosphate dehydrogenase (GAPDH), the mRNA level of target genes was calculated using a 2^−ΔΔCT^ method.

### Western blotting

Cells and tissues were lysed to release total proteins for quantification. Then, samples were loaded onto sodium dodecyl sulfate-polyacrylamide gel electrophoresis to separate proteins, which were then transferred to polyvinylidene fluoride membranes (FFP24; Beyotime). Membranes were incubated overnight with primary antibodies (anti-WDR3, 1:1000, PA5-144030, Thermo; anti-GAPDH, 1:2500, ab181602, Abcam). After washing, membranes were incubated for 1 h with a 2000-fold diluted secondary antibody (Goat Anti-Rabbit IgG H&L, ab6721, Abcam). Finally, membranes were developed using enhanced chemiluminescence solution (P1000, APPLYGEN), followed by the scanning of exposed films. ImageJ software was used to quantify the band intensities, and the protein expression of WDR3 relative to GAPDH was calculated.

### Cell counting kit-8 (CCK-8)

Transfected or Nilotinib-treated U2-OS cells were inoculated in 96-well plates (2000 cells per well). After 24 h of incubation (37 ℃, 5% CO_2_), each well was supplemented with 10 µL of CCK-8 reaction solution (C0037, Beyotime). Two hours later, the optical density of each well at 450 nm was measured using a microplate reader (DR-3518G, Wuxi Hiwell Diatek) to evaluate the cell viability.

### Transwell assay

The Transwell assay was conducted to assess the cell migration and invasion. For migration assays, digested U2-OS cells were seeded into Transwell chambers, which was then cultured in the lower chamber containing medium for 24 h culture under standard conditions. Afterwards, the Transwell chamber was fetched out to remove medium. After undergoing washing and fixation, crystal violet (C0121, Beyotime) was added to the chambers for 20 min to visualize non-migrated cells. For invasion assays, the bottom of the Transwell chambers was covered with 50 mg/L Matrigel (354234, Corning) diluted at 1:4 until it polymerized into a gel. Cell culture, fixation, and staining were performed as described for migration assays. Cell migration and invasion were observed in three randomly selected fields under a microscope.

### Animals

Thirty male Balb/c nude mice (5–6 weeks old) were purchased from the Experimental Animal Center of Yangzhou University, where they were housed under a 12-hour light/dark cycle and had free access to adequate food and water. Of which, 18 mice were randomly divided into three groups (OS, OS + sh-NC, and OS + sh-WDR3; six mice per group) and injected subcutaneously in the right axilla with 1 × 10^7^ U2-OS, sh-NC-transfected U2-OS, and sh-WDR3-transfected U2-OS cells, respectively, to induce xenograft tumor formation. Body wrights and tumor volumes were recorded every seven days over a four-week period. On day 28th, mice were anesthetized with 4% isoflurane (HY-A0134, MCE) and then euthanized to collect tumor tissues.

Another 12 mice were randomized into OS and OS + Nilotinib (OS + NIL) groups (*n* = 6) and injected subcutaneously with 1 × 10^7^ U2-OS cells to constructed the xenograft model of osteosarcoma. One week later, Nilotinib was diluted in dimethyl sulfoxide (D8371, Solarbio) and administered via oral gavage to mice in the OS + NIL group (30 mg/kg) using a carrier containing 0.5% hydroxypropyl methylcellulose (HPMC, HY-A0104J, MCE) and 0.05% Tween80 (TB360, Solarbio). The OS group received an equal dose of 0.5% hydroxypropyl methylcellulose and 0.05% Tween80 via gavage. After daily gavage treatment for three weeks, the mice were euthanized to collect tumor samples and lung tissues. All animals were carried out with the approval of the Experimental Animal Ethics Committee of Yangzhou University (No.202312013).

### Immunohistochemical

Tumor tissues from each group of mice were prepared into sections and incubated overnight with a 1000-fold dilution of antibodies against WDR3 (PA5-144030, Thermo) and Ki67 (9129 S, Cell Signaling). The washed sections were incubated with a secondary antibody (Goat Anti-Rabbit IgG H&L, ab6721, Abcam) for 15 min at a dilution of 1:2000. To visualize the target proteins, the sections were stained with DAB staining solution (P0202, Beyotime) for 30 min, followed by counterstaining with hematoxylin (G1080, Solarbio) for 3 min. After cleaning, the sections were dehydrated, mounted, and photographed under a microscope.

### Terminal Deoxynucleotidyl transferase (TdT)-mediated dUTP nick-end labelling (TUNEL)

The prepared tumor tissue sections were routinely dewaxed and hydrated. Following the instructions of TUNEL kit (C1091, Beyotime), 50 µL of the configured proteinase K working solution was added dropwise to the sections for digestion of 30 min, followed by the incubation with a mixture of 5µL TdT enzyme, 45µL fluorescent labeling solution, and 50µL TUNEL test solution for 30 min. After washing, the sections were stained with 4’,6-diamidino-2-phenylindole (DAPI, C1005, Beyotime) for 10 min. Ultimately, the sections were mounted with antifade mounting medium (p0126, Beyotime) and observed under a microscope.

### Recombinant protein expression and purification

The WDR3 gene was cloned into the pET-28b (+) expression vector (with GFP and His tags) and transformed into *E. coli* strain BL21 (DE3). The transformed strains were cultured in LB liquid medium containing 100 µg/mL Ampicillin (ST008, Beyotime) at 37 °C and 170 rpm until the OD600 of the bacterial culture reached 0.6–1.0. The addition of isopropyl-beta-D-thiogalactoside (ST098, Beyotime) to a final concentration of 1mM induced the protein expression at 37℃ for 4 h. Afterwards, the bacterial culture was centrifuged at 15,000 g for 1 min at 4 °C to collect the precipitate. The harvested bacterial pellet was resuspended and lysed by sonication on ice. Then, the recombinant proteins were purified using BeyoGold™ His-tag Purification Resin (P2233, Beyotime). Finally, the proteins were eluted on an elution column and concentrated by ultrafiltration.

To construct protein mutants, the IDRs of WDR3 were predicted on the IUPred2A website. These predictions guided the construction of three WDR3 IDR mutants (WDR3-MUT1, WDR3-MUT2, and WDR3-MUT3). A fusion mutant incorporating the IDR of hnRNPA1, known to drive condensate formation, were synthesized to create a rescue phase-separated WDR3 mutant (MUT-IDR). WDR3-MUT and MUT-IDR were then cloned into pET-28b (+) expression vectors (with GFP and His tags) to induce protein expression, respectively. The procedures for protein expression and purification are described above.

### Droplet formation assay

In this study, 20 µM WDR3-GFP purified protein was added to a buffer containing 10% PEG8000 (81268, Sigma) and NaCl (ST1641, Beyotime) with different concentrations (75 mM, 150 mM, 300 mM). The protein solution was then loaded onto a slide and condensates with liquid-like behavior were observed under a fluorescence microscopy. The same method was also applied to observe the droplet formation of purified WDR3-GFP protein in 125 mM NaCl (pH7.5) and 10%PEG buffer at different concentrations (10 µM, 20 µM, 40 µM).

To investigate the intracellular droplet formation, lentiviral expression vectors for WDR3-MUT and MUT-IDR were constructed and transfected into U2-OS cells. Cells were seeded and cultured overnight until wall attachment. The droplet formation in the cells was observed under a confocal laser scanning microscope (CLSM, TCS SP8, Leica).

### Fluorescence recovery after photobleaching (FRAP)

To further verify the recovery of fluorescence activity of WDR3 after photobleaching, 20 µM WDR3-GFP purified proteins were added in a buffer containing 10% PEG8000 and 125 mM NaCl (pH7.5) to form droplets and then subjected to FRAP assay using CLSM. The FRAP assay was programmed with a 488 nm 100% power laser, a 1.5 μm radius area as the target focus, and a bleaching time of 20 s. The recovery of protein condensates was photographed at 40 s and 60 s post-photobleaching. The same procedures were applied to detect phase separation of exogenous WDR3 in WDR3-GFP-, WDR3-MUT-, or MUT-IDR-transfected U2-OS cells.

### Immunofluorescence

In this study, immunofluorescence was used to examine the formation of endogenous and exogenous WDR3 phase-separated condensates in cells. To facilitate observation, cells were sequentially fixed, permeabilized, blocked, and incubated in 200 µL of anti-WDR3 anbtibody (9129S, Cell Signaling) diluted at 1:200 overnight. The following day, cells were incubated in a 1:500 diluted mixture of IgG H&L and DAPI for 30–60 min. After mounting the slices, the fluorescence of each group was detected under a CLSM.

### Hematoxylin-eosin (HE) staining

In this study, lung tissues were prepared into sections and processed for HE staining according to the manufacturer’s protocol (C0105S, Beyotime). Briefly, the sections were dewaxed and rehydrated, followed by staining with hematoxylin for 5 min and eosin for 1–2 min. After dehydration with alcohol (10009218, SINOPHARM), the sections were cleared in xylene (10023418, SINOPHARM) for 3 min. Finally, the number of metastatic lung nodules was observed and counted under a microscope.

### Statistical analysis

All data were presented as mean ± standard deviation and processed on GraphPad 10.1.2. Comparisons between two groups were conducted using unpaired *t* tests, while comparisons among multiple groups were performed using one-way analysis of variance (ANOVA) with Tukey’s post hoc test. Differences between groups over continuous time were compared using two-way repeated measures ANOVA. A *P*-value below 0.05 was defined as statistical significance.

## Results

### Predictive efficiency of LLPS-related genes in osteosarcoma prognosis

**Fig. 1:**
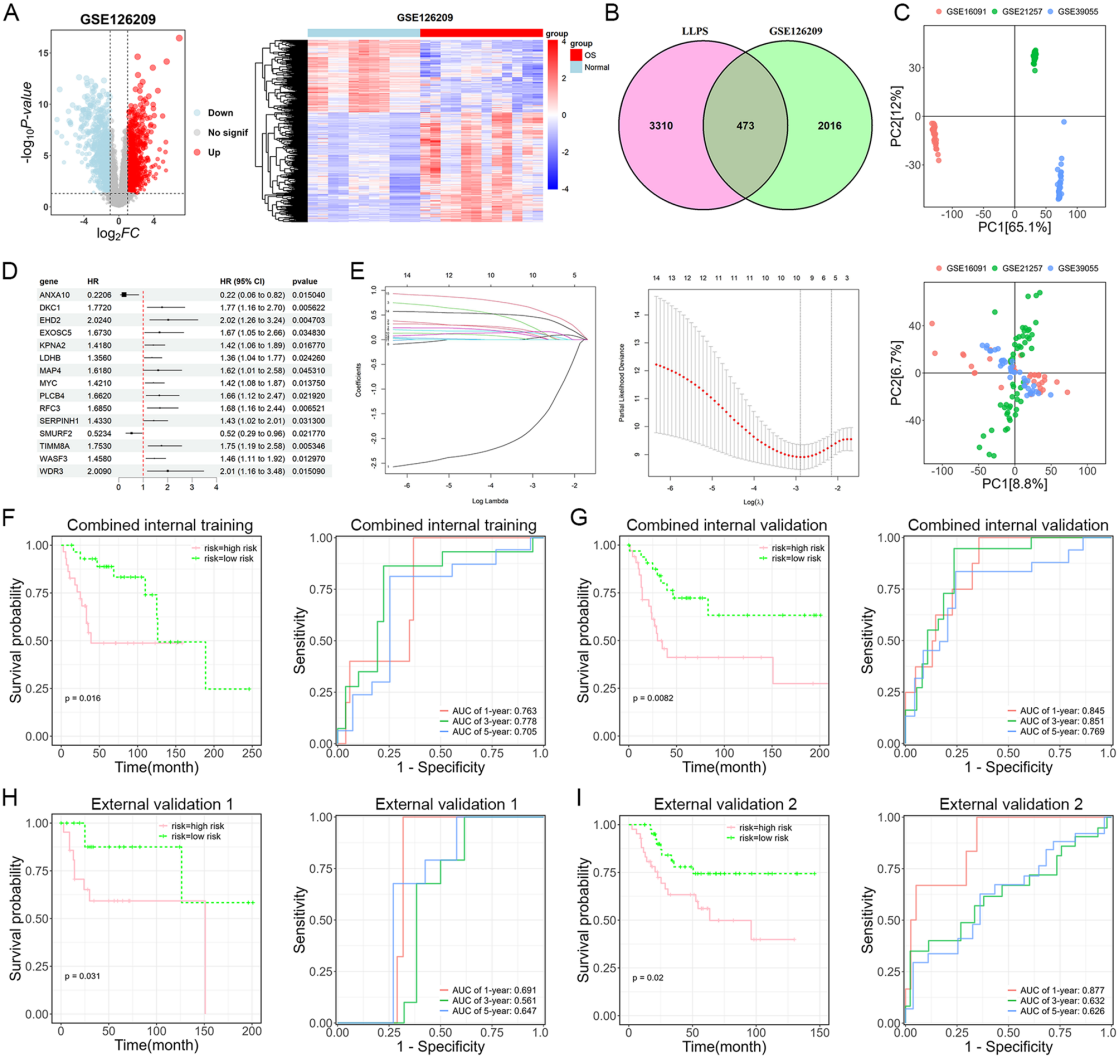
Screening of prognostic biomarkers to predict osteosarcoma survival based on a risk score-based model. (**A**) The volcano plot (left panel) and heatmap (right panel) showing 1497 DEGs between osteosarcoma and normal controls. (**B**) The Venn diagram depicts 473 intersected genes between DEGs and LLPS-related genes. (**C**) PCA plots before (top panel) and after (bottom panel) batch effect removal. (**D**) The univariate Cox regression analyses screened out 15 genes related to osteosarcoma prognosis. (**E**) Coefficients of the LASSO analysis (left panel) and partial likelihood deviance analysis (right panel) on 15 genes. (**F**–**I**) The KM (left panel) and ROC (right panel) curves of the combined internal training set (**F**), combined internal validation set (**G**), external validation cohort 1 (**H**), and external validation cohort 2 (**I**)

Based on the expression profiles of the GSE126209 dataset, this study identified 2489 DEGs between osteosarcoma and normal samples, with 1497 DEGs up-regulated and 992 DEGs down-regulated in osteosarcoma (Fig. 1A). Among these DEGs, 473 of them belonged to LLPS-related genes (Fig. 1B). To screen prognostically relevant genes, the GSE16091, GSE21257, and GSE39055 datasets were merged. After removing the batch effect, the samples were evenly distributed without significant discrete clusters (Fig. 1C). In the combined dataset, 15 genes significantly associated with survival time in osteosarcoma (log-rank test, *P* < 0.05) were identified using the univariate Cox regression algorithm (Fig. 1D). Among these 15 genes, only ANXA10 and SMURF2 were found to be prognostic protective factors for osteosarcoma. LASSO was then carried out to select models with excellent performance but the minimal number of variables (Fig. 1E). With the optimal λ value, a gene list comprising ANXA10, MYC, TIMM8A, WASF3, and WDR3 was identified as prognostic biomarkers to establish a predictive system according to the equation: Risk Score = -0.651*ANXA10 + 0.974*MYC + 0.051*TIMM8A + 0.234*WASF3 + 0.347*WDR3. The samples were categorized into high- and low-risk groups for comparison based on the median risk score. In both the internal training and validation sets, patients in the high-risk group had significantly lower survival rates than those in the low-risk group, with the area under curve (AUC) values of 1-, 3-, and 5-year ROCs all exceeding 0.7 (Fig. 1F-G). To further assess the predictive efficacy of the model, we conducted validation in two independent external cohorts. The results confirmed that patients in the high-risk group had a significantly lower probability of survival than those in the low-risk group, with the predictive model demonstrating high sensitivity and specificity (Fig. 1H-I).

### Characteristic evaluation of prognostic biomarkers

**Fig. 2:**
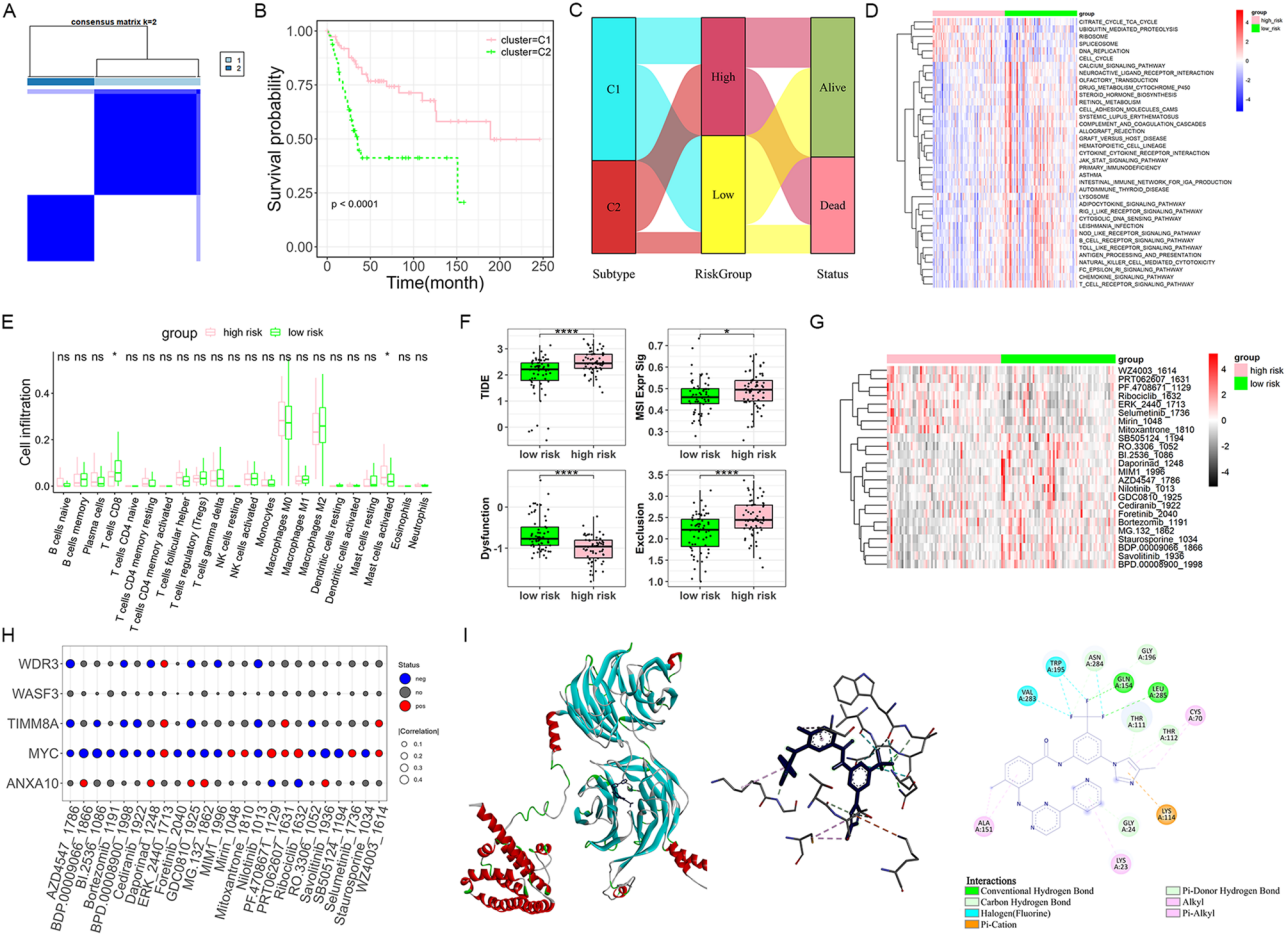
Characteristic evaluation of prognostic biomarkers. (**A**) Consensus matrix heatmap defining two clusters (k = 2) and their correlation areas. (**B**) KM curves showing the survival difference between high- and low-risk groups. (**C**) Sankey diagram exhibiting the distribution of two clusters in risk groups. (**D**–**G**) Differences in GSVA pathway scores (**D**), immune cell infiltrations (**E**), TIDE scores (**F**), and drug IC50 values (**G**) between high- and low-risk groups. *P < 0.05; ****P < 0.0001. (**H**) Correlations between prognostic biomarkers and drug IC50 values. (**I**) Molecular docking showing the interaction of WDR3 and Nilotinib

Thereafter, this study performed a multidimensional comparison of differences between high- and low-risk groups. First, molecular subtypes of osteosarcoma were identified using consensus clustering analysis. The results showed that k = 2 appeared to be the best choice for grouping the samples into cluster 1 and 2 (Fig. 2A). Patients in cluster 1 demonstrated a more favorable prognosis compared to those in cluster 2 (Fig. 2B), and were more commonly distributed in the low-risk group, which was prone to the status of being alive (Fig. 2C). The limma package also identified 37 pathways with differences in GSVA scores between high- and low-risk groups (Fig. 2D). The majority of these pathways was significantly positively correlated with ANXA10, but markedly negatively correlated with MYC, TIMM8A, and WDR3 (Supplementary Fig. 1A). There were also significant differences in infiltration levels of CD8 T cells and activated mast cells between risk groups (Fig. 2E). Among them, the infiltration level of CD8 T cells was significantly negatively correlated with MYC expression, while the activated mast cells were positively correlated with MYC and WDR3 (Supplementary Fig. 1B). TIDE and MSI scores can predict the immune evasion potential for patients with cancer and their response to immunotherapy. In this study, patients in the low-risk group exhibited significantly lower TIDE and MSI scores than those in the high-risk group, suggesting a reduced potential of tumor immune escape and an enhanced likelihood of response to immunotherapy (Fig. 2F).

To assess the sensitivity of each sample to various drugs, the oncoPredict tool was applied. Upon comparison between the high- and low-risk groups, we observed significant differences in sensitivity for a total of 24 drugs (Fig. 2G). The sensitivity to these drugs was significantly correlated with the expression of MYC, but had no significant association with WASF3 (Fig. 2H). Moreover, the expression of WDR3 may affect the sensitivity of patients with osteosarcoma to drugs, such as AZD4547 and Nilotinib (Fig. 2H). To probe into the possible binding of proteins encoded by five prognostic genes with 24 drugs, molecular docking was conducted and 100 valid docking results were obtained. Among them, the interaction of WDR3 and Nilotinib depicted the lowest binding energy (-9.3 kcal/mol), with hydrogen bond on residues such as GLN-154 and LEU-285 contributing to the stability of the receptor-ligand complex (Fig. 2I). Nilotinib was also bound to WDR3 residues by Alkyl, Halogen, Pi-Alkyl, and Pi-Cation interactions (Fig. 2I).

### Effect of prognostic biomarker WDR3 on the progression of osteosarcoma in vitro and in vivo

**Fig. 3:**
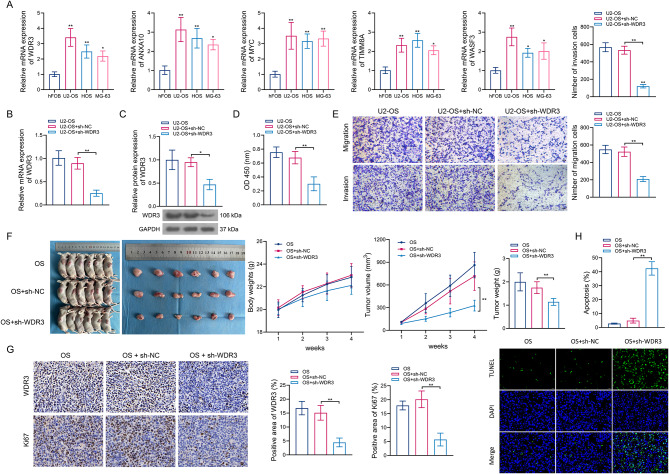
Effect of WDR3 downregulation on the progression of osteosarcoma in vitro and in vivo. (**A**) Expression validation of five prognostic biomarkers using qPCR. (**B**–**C**) The mRNA (**B**) and protein (**C**) expression of WDR3 in U2-OS cells transfected with sh-NC or sh-WDR3. (**D**–**E**) Effects of WDR3 downregulation on the cell proliferation (**D**), as well as cell migration and invasion (**E**). (**F**) Body weight, tumor value and tumor weight of mice injected with sh-NC- or sh-WDR3-transfected U2-OS cells. (**G**–**H**) Effects of WDR3 downregulation on Ki67 expression (**G**) and tumor cell apoptosis (**H**). *P < 0.05; **P < 0.01

Compared with normal osteoblastic hFOB, this study validated the expression patterns of five prognostic biomarkers in three osteosarcoma cell lines (U2-OS, HOS, and MG-63). qPCR results suggested that the candidate phase separation-related genes were all up-regulated in osteosarcoma cells (Fig. 3A), which was consistent with the results of bioinformatic analyses. Considering the binding stability of WDR3 to Nilotinib, this study focused on exploring the impact of WDR3 phase separation on osteosarcoma and its involvement in Nilotinib-based antitumor therapy. First, we used lentiviral transfection to specifically down-regulate WDR3 expression in U2-OS cells to observe its effect on osteosarcoma progression in vitro and in vivo. As expected, sh-WDR3 significantly reduced WDR3 mRNA and protein expression levels in U2-OS cells compared with those transfected with sh-NC (Fig. 3B–C). Meanwhile, U2-OS cells transfected with sh-WDR3 demonstrated a significant inhibition of cell viability (Fig. 3D), as well as cell migration and invasion (Fig. 3E), compared with their negative controls. In vivo, downregulation of WDR3 also significantly reduced the tumor volume and tumor weight in xenografted mice with osteosarcoma, without affecting their body weights (Fig. 3F). In addition, sh-WDR3 transfection significantly reduced the positive area of WDR3 and Ki67 in mice tumor tissues (Fig. 3G), while promoting apoptosis (Fig. 3H), relative to controls. Thus, silencing of WDR3 can effectively alleviate the malignant progression of osteosarcoma.

### WDR3 exhibited phase-separated condensates with liquid-like behavior in OS cells

**Fig. 4:**
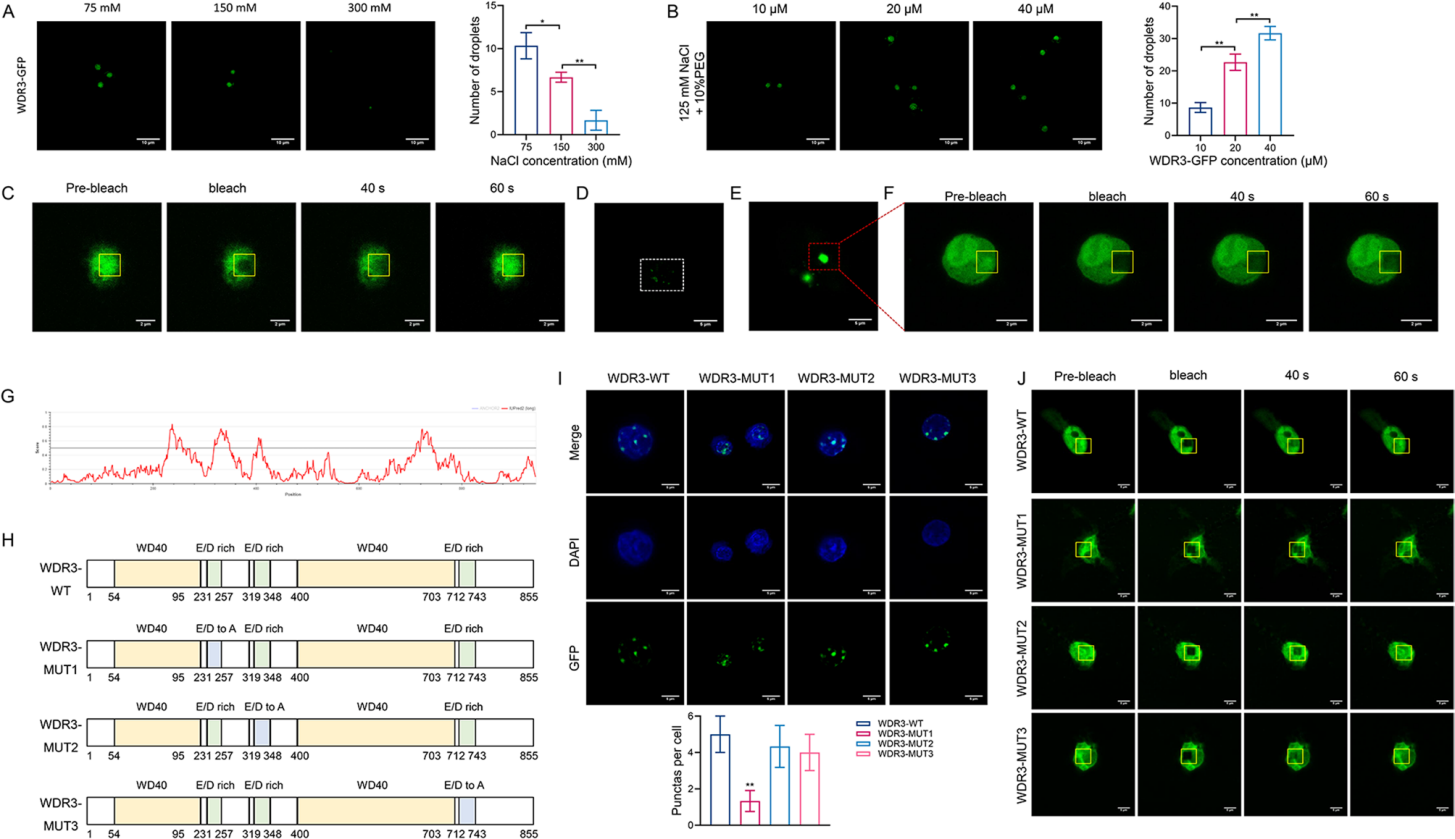
WDR3 exhibited phase-separated condensates with liquid-like behavior in OS cells. (**A**–**B**) The droplet formation of WDR3 under different concentrations of NaCl (**A**) and WDR3-GFP recombinant protein (**B**). *P < 0.05; **P < 0.01. (**C**) FRAP of WDR3-GFP droplets. (**D**) The droplet formation of endogenous WDR3 in U2-OS cells. (**E**) Phase-separated condensates of exogenous WDR3-GFP protein in U2-OS cells. (**F**) FRAP of WDR3-GFP protein exogenously transfected in U2-OS cells. (**G**) IDR of WDR3 predicted using the IUPred2A online tool. (**H**) Domain structure of WDR3-WT and three WDR3 IDR mutants. (**I**) Effect of three WDR3 IDR mutants on the number of intracellular WDR3 droplets. **P < 0.01 versus WDR3-WT. (**J**) Effect of three WDR3 IDR mutants on the recovery of WDR3 condensate after photobleaching

The droplet formation experiments showed that WDR3 can form circular condensates in the presence of low concentration of salt solution, while the number of droplets significantly reduced with increasing NaCl concentration (Fig. 4A). Additionally, the number of droplets increased with higher concentration of the WDR3-GFP recombinant protein (Fig. 4B). After photobleaching, the fluorescence signals in the target field were weakened but rapidly recovered within 60 s, suggesting the liquid-like behavior within the droplets (Fig. 4C). In U2-OS cells, the presence of endogenous WDR3 were observed by immunofluorescence (Fig. 4D). Furthermore, U2-OS cells exogenously transfected with WDR3-GFP demonstrated stronger fluorescence and larger WDR3 condensates of (Fig. 4E). Photobleaching of this region also resulted in gradual recovery of the WDR3 condensate fluorescence signal (Fig. 4F), further confirming that WDR3 can form droplets through phase separation.

To further investigate the phase-separation ability of WDR3, we mutated its IDR, where amino acid mutations have been shown to alter the phase-separation properties of proteins thereby impairing their recombination and reprogramming [28]. Using the IUPred2A online tool, this study predicted that the IDRs of WDR3 were mainly concentrated in the region of amino acids 231–257, 319–348, and 712–743, which may contribute to the formation of dimers or multimers (Fig. 4G). Furthermore, all glutamate and aspartate residues within these three regions were substituted with alanine (Supplementary Fig. 2), guiding the construction of three WDR3 IDR mutants, designated as WDR3-MUT1, WDR3-MUT2, and WDR3-MUT3 (Fig. 4H). Among these, only WDR-MUT1 significantly reduced the number of intracellular WDR3 droplets (Fig. 4I) and slowed the reformation of WDR3 condensates after photobleaching (Fig. 4J), prompting its selection for subsequent investigations.

### Phase separation of WDR3 expedited OS metastasis in vitro

**Fig. 5:**
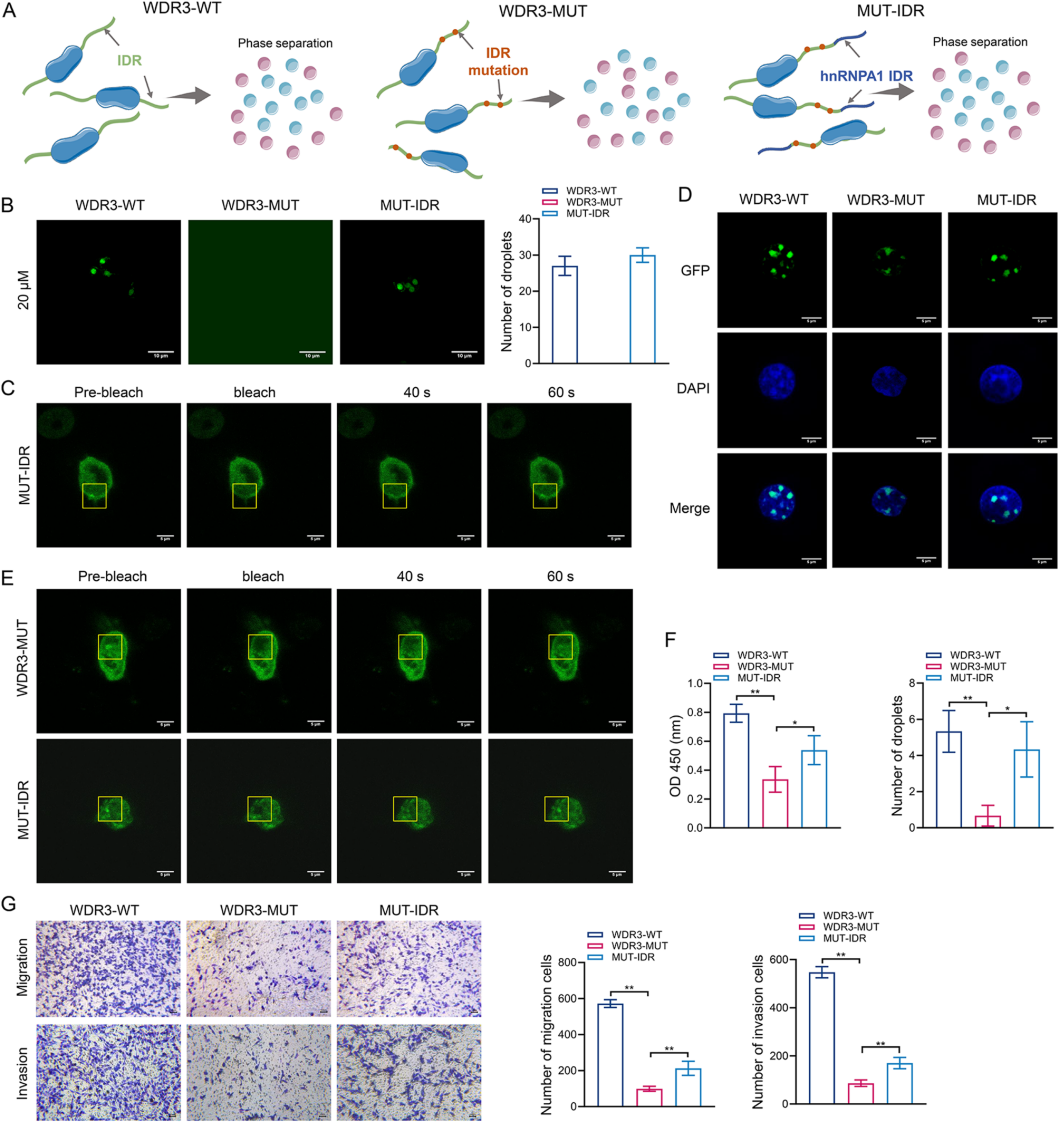
Phase separation of WDR3 expedited OS metastasis in vitro. (**A**) Schematic illustration of WDR3 mutation and rescue of phase separation. (**B**) The droplet formation of WDR3-WT, WDR3-MUT, and MUT-IDR. (**C**) FRAP of MUT-IDR. (**D**) The droplet formation of WDR3 in U2-OS cells transfected with WDR3-WT, WDR3-MUT, and MUT-IDR. (**E**) FRAP of WDR3-MUT and MUT-IDR in U2-OS cells. (**F**–**G**) The proliferation (**F**) and metastatic ability (**G**) of U2-OS cells transfected with WDR3-WT, WDR3-MUT, and MUT-IDR. *P < 0.05; **P < 0.01

The IDR of hnRNPA1 facilitates protein assembly and droplet formation [29], and thus can be fused to WDR3-MUT to rescue the phase separation of WDR3 (Fig. 5A). Under the microscope, WDR3-MUT was found to disrupt the droplet-forming ability of WDR3, resulting in dispersed fluorescence and an inability to aggregate. This dysfunction was then rescued by the fusion of hnRNPA1 IDR, allowing WDR3 to reaggregate and form droplets (Fig. 5B). After photobleaching the target region of MUT-IDR, the fluorescence intensity of WDR3 was gradually restored and the droplet aggregation ability was rescued (Fig. 5C).

Following the transfection of U2-OS cells with WDR3-WT, WDR3-MUT, and MUT-IDR, changes in WDR3 phase-separation characteristics were also investigated in this study. As expected, the fluorescence in the WDR3-MUT group began to disperse, accompanied by a decrease in the number of droplets. However, the droplets in the MUT-IDR group were reconcentrated and the number of droplets recovered significantly (Fig. 5D). FRAP results indicated that the fluorescence in the photobleached regions of WDR3-MUT-transfected cells remained unchanged, which could be recovered by the transfection of MUT-IDR (Fig. 5E). More importantly, mutation of WDR3 significantly down-regulated the proliferation and metastatic ability of U2-OS cells, but the transfection of MUT-IDR restored the malignant phenotype of tumor cells (Fig. 5F–G). Thus, the phase separation of WDR3 could promote the proliferation, migration, and invasion of osteosarcoma in vitro.

### Nilotinib mitigated osteosarcoma progression via inhibition of WDR3 phase separation

**Fig. 6:**
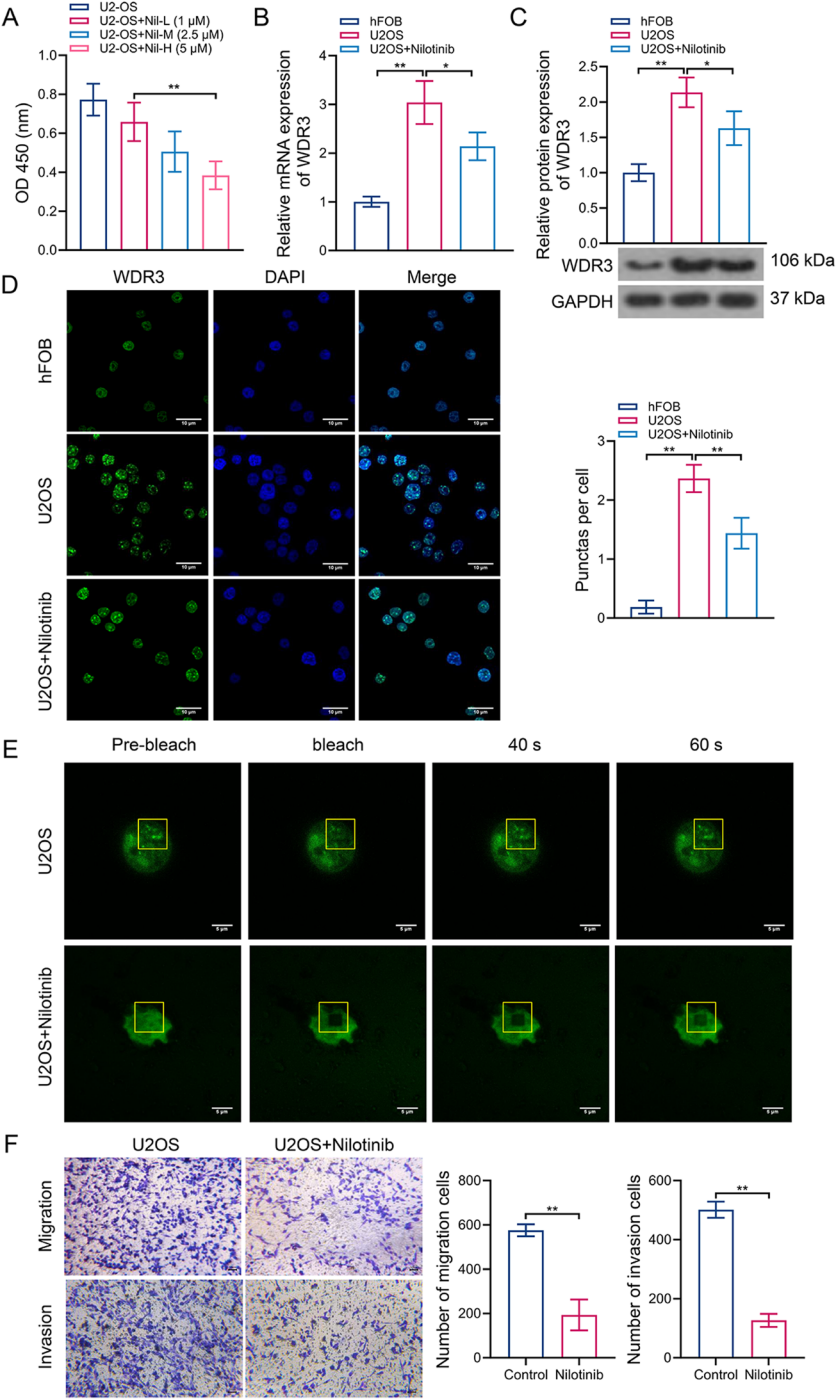
Effect of Nilotinib treatment on WDR3 phase separation and tumor metastasis *in vitro.* (**A**) Changes of cell proliferation under the treatment of Nilotinib with different concentrations. (**B**–**C**) Effects of Nilotinib treatment on WDR3 mRNA (**B**) and protein (**C**) expression. (**D**) The droplet formation of WDR3 in U2-OS cells treated with Nilotinib. (**E**) FRAP of WDR3 droplets in U2-OS cells treated with Nilotinib. (**F**) Effect of Nilotinib treatment on migration and invasion of U2-OS cells. *P < 0.05; **P < 0.01

To clarify the therapeutic mechanism of Nilotinib on osteosarcoma, we treated U2-OS cells with different concentrations of Nilotinib in vitro for 24 h. These treated cells were significantly reduced in proliferation in a dose-dependent manner (Fig. 6A). Nilotinib treatment also significantly reduced the mRNA and protein expression levels of WDR3 in U2-OS cells (Fig. 6B–C). Notably, the fluorescence intensity of WDR3 and punctas per cell were significantly increased in U2-OS cells compared to hFOB cells, whereas Nilotinib treatment significantly suppressed these abnormalities (Fig. 6D). The FRAP results suggested that the fluorescence intensity of WDR3 condensates in untreated U2-OS cells after photobleaching could recover within 60 s, which was not observed in Nilotinib-treated cells (Fig. 6E). Moreover, the addition of Nilotinib significantly reduced the migration and invasion abilities of U2-OS cells (Fig. 6F).

**Fig. 7:**
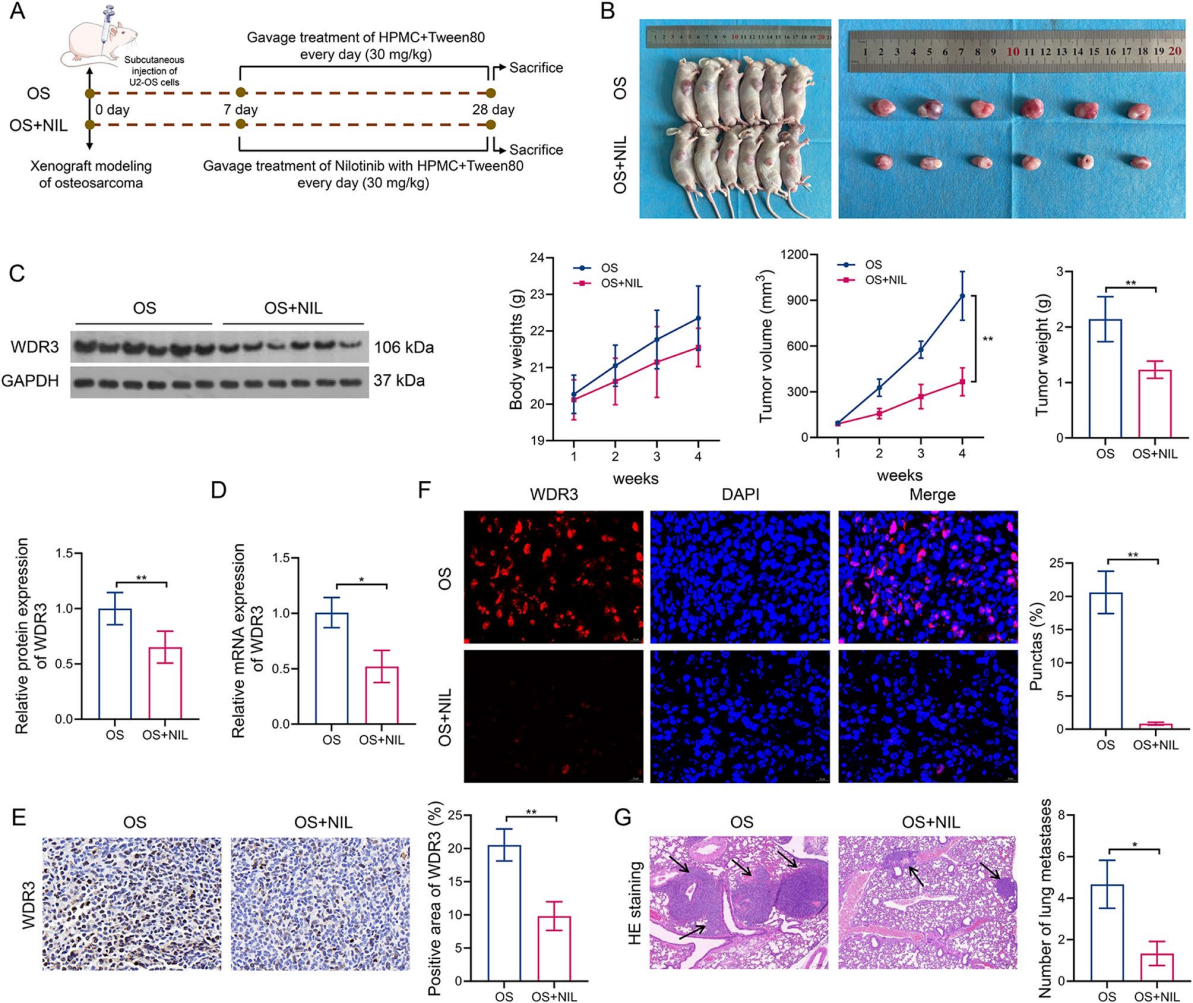
Effects of Nilotinib treatment on WDR3 phase separation and tumor progression *in vivo.* (**A**) The schedule for animal experiments. (**B**) Body weight, tumor volume, and tumor weight in xenograft mice of osteosarcoma with or without Nilotinib treatment. (**C**–**D**) The protein (**C**) and mRNA (**D**) expression of WDR3 in mice treated with/without Nilotinib. (**E**) The positive area of WDR3 in tissues of mice treated with/without Nilotinib. (**F**) Effects of in vivo treatment of Nilotinib on the number of WDR3 condensates. (**G**) The number of pulmonary metastatic nodules in each group of mice. *P < 0.05; **P < 0.01

The animal experiments were also carried out to confirm the role of WDR3 phase separation in the Nilotinib-based treatment for osteosarcoma. The schedule for animal experiments is shown in Fig. 7A. Consistent with the above results, the treatment of Nilotinib significantly reduced the tumor volume and tumor weight in xenografted mice of osteosarcoma, but unaffected the body weight of mice (Fig. 7B). Meanwhile, continuous Nilotinib treatment also significantly down-regulated the mRNA and protein expression levels of WDR3, as well as reduced the positive area of WDR3, compared to untreated mice (Fig. 7C–E). Immunofluorescence showed that the number of WDR3 condensates decreased significantly under the treatment of Nilotinib (Fig. 7F), suggesting a downregulation of the phase separation level of WDR3. Moreover, mice in the treatment group exhibited a significantly reduced number of pulmonary metastatic nodules compared to the control group (Fig. 7G). Therefore, Nilotinib may alleviate the progression of osteosarcoma in vivo and in vitro by inhibiting the phase separation of WDR3.

## Discussion

The challenges in extending the survival of patients with metastatic osteosarcoma have prompted the quest for multiple prognostic biomarkers. Commencing with the LLPS mechanism, this study identified five LLPS-related genes (ANXA10, MYC, TIMM8A, WASF3, and WDR3) with prognostic potentials through bioinformatics approaches, and the corresponding risk model constructed was capable of predicting the survival likelihood of patients with osteosarcoma. These prognostic biomarkers were significantly associated with immune cell infiltration, tumor immune escape, and drug sensitivity. Among them, WDR3 and Nilotinib demonstrated optimal binding stability in molecular docking models. WDR3 not only serves as a prognostic risk factor for osteosarcoma, but is also highly expressed in U2-OS cells. Functional experiments have shown that the knockdown of WDR3 inhibited the proliferation and metastatic ability of osteosarcoma cells while suppressing tumor growth. More importantly, WDR3 can form condensates with liquid-like behavior in U2-OS cells, and mutation of its IDR can eliminate the phase-separated level of WDR, thus reversing the aggressive phenotype of osteosarcoma cells.

WDR3, also known as DIP2 and UTP12, belongs to a family of eukaryotic genes that carry the WD repeat region [30]. It encodes a 943 amino acid nuclear protein composed of a 10 WD repeat sequence module localized to human chromosome 1p12-p13 [31]. Su et al. found that the overexpression of WDR3 is associated with low survival in cancer and promotes pancreatic cancer proliferation and invasion by interacting with GATA4 to activate the Hippo pathway [32]. In thyroid cancer, WDR3 maintains genomic stability in patients, while its location on the 1p12 chromosome contributes to the disease susceptibility [33, 34]. WDR3 expression is linked to inflammatory mediators and can be reduced following the treatment with anti-inflammatory drugs [35]. McMahon et al. proposed that WDR3 deficiency leads to ribosome biogenesis defects by affecting 18s rRNA processing, thereby reducing p53-mediated proliferation of cancer cells [36]. Although the role of WDR3 in osteosarcoma remains unexplored, it has been found to promote cancer stem cell characteristics by inhibiting USF2-mediated RASSF1A transcription [37]. In a multicenter case-control study, genetic polymorphisms in RASSF1A were found to be associated with the risk of osteosarcoma and metastasis in young Chinese adults [38]. As a tumor suppressor, RASSF1A exerts anticancer effects by regulating the Wnt/β-catenin pathway [39]. These findings provide mechanistic support for our observation that WDR3 may promote osteosarcoma metastasis by inhibiting the transcriptional regulation of RASSF1A. Furthermore, our results indicated that WDR3 expression was significantly positively correlated with the spliceosome pathway score, but had negative correlations with the lysosome pathway. SNRPB, a core component of the spliceosome, has been shown to induce malignant behavior in osteosarcoma [40]. C1GALT1 enhances the drug resistance and metastatic propensity of osteosarcoma by promoting lysosomal degradation and effluence [41]. Therefore, we speculated that WDR3 phase separation-mediated tumorigenesis in osteosarcoma may be associated with the coordinated regulation of SNRPB expression and lysosomal degradation pathways.

**Fig. 8:**
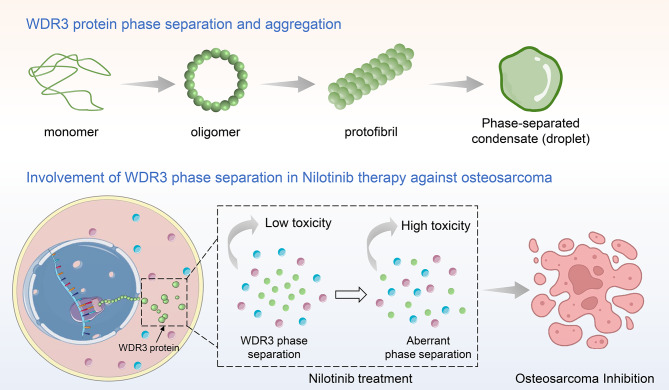
Schematic illustration for the mediation of WDR3 phase separation in the treatment of Nilotinib against osteosarcoma metastasis

Regarding therapeutic implications, WDR3 bound stably to Nilotinib in this study and mediated the therapeutic mechanism of Nilotinib against osteosarcoma. As a second-generation tyrosine kinase inhibitor that allows for a superior deep molecular response, Nilotinib is approved for the first-line treatment of BCR-ABL-positive chronic granulocytic leukemia [42]. Beyond hematologic malignancies, Nilotinib has demonstrated efficacy in gastrointestinal stromal tumors harboring KIT exon 11 mutations [43]. By targeting the suppression of DDR1, Nilotinib effectively blocked the migration of breast cancer cells [44]. Nilotinib has also been reported to improve outcomes in patients with colorectal cancer receiving anti-PDL1 therapy by restoring MHC-I expression [45]. In osteosarcoma, Nilotinib downregulates the expression of prognostic marker MAPK1 to induce apoptosis [46]. Of particular relevance, Wei et al. suggested that Nilotinib may exert therapeutic benefits through the modulation of phase separation in cells [47]. Consistent with this hypothesis, our study provides direct evidence that Nilotinib significantly inhibited the production of phase-separated condensates of WDR3, thereby suppressing tumor growth and metastasis in vivo and in vitro (as shown in Fig. 8). Furthermore, WDR3 expression was positively correlated with the infiltration level of activated mast cells in osteosarcoma. In rat peritoneal mast cells, Nilotinib decreased the expression of pro-inflammatory cytokines and TNF-α and dose-dependent decreased histamine release from mast cells [48]. Mast cell infiltration is common in the inflammatory response to malignant osteosarcoma, which occurs mainly at the tumor margin and may lead to osteolysis and tumor invasion, but facilitates immunomodulatory therapy [49]. It was also reported that mast cell accumulation in osteosarcoma is regulated by the CXCL6-CXCR2 axis [50]. Therefore, disruption of WDR3 phase separation is closely associated with mast cell-mediated inflammatory responses, thus affecting the mechanisms of metastasis and drug resistance in osteosarcoma.

The regulation of LLPS represents a highly complex biological process involving not only crosstalk between intracellular components but also extracellular environmental factors such as temperature, ionic concentration, and pH [51], which may further affect the phase-separation characteristics of proteins. In addition, the cellular energetic state including APT levels may affect the kinetic changes of LLPS [52]. However, these factors that may affect the phase separation level of WDR3 were not discussed, which is one of the limitations of our study. Furthermore, whether the phenomenon of phase separation is regulated by WDR3-interacting proteins remains unknown. Finally, Nilotinib is associated with cardiovascular adverse events in clinical use [53], but the cardiovascular toxicity of Nilotinib was not systematically explored in this study. Therefore, further studies are recommended to focus on exploring the intracellular mechanisms affecting WDR3 phase separation, as well as the resistance and toxicity of Nilotinib in osteosarcoma treatment.

## Conclusion

This study proposed five LLPS-related biomarkers with the potential to predict the survival of osteosarcoma. Among them, WDR3 emerged as a prognostic risk factor for osteosarcoma and bound stably to Nilotinib in molecular docking models. Functionally, WDR3 was significantly overexpressed in osteosarcoma cells, whereas its downregulation inhibited the malignant progression of osteosarcoma both in vivo and in vitro. In addition, WDR3 could form droplets, and its IDR mutation eliminated phase-separated levels of WDR3, thereby ameliorating the aggressive phenotype of osteosarcoma cells. Nilotinib may also reduce WDR3 phase-separated condensates and inhibit tumor growth and metastasis.

## Electronic supplementary material

Below is the link to the electronic supplementary material.

Supplementary Material 1

Supplementary Material 2

Supplementary Material 3

## Data Availability

No datasets were generated or analysed during the current study.

## Abbreviations

ANOVA: One-way analysis of variance
CCK-8: Cell counting kit-8
CDF: Cumulative distribution function
DAPI-4’,6: Diamidino-2-phenylindole
DEGs: Differentially expressed genes
FRAP: Fluorescence recovery after photobleaching
GEO: Gene Expression Omnibus
IDR: Intrinsically disordered region
LASSO: Least absolute shrinkage and selection operator
LLPS: Liquid-liquid phase separation
MSI: Microsatellite instability
PCA: Principal component analysis
qPCR: Quantitative real-time polymerase chain reaction
ROC: Receiver operating characteristic
TdT: Terminal deoxynucleotidyl transferase
TUNEL: TdT-mediated dUTP nick-end labelling
