# Supplementary material for: WDR3 undergoes phase separation to mediate the therapeutic mechanism of Nilotinib against osteosarcoma
Source: J Exp Clin Cancer Res. 2025 Jul 11;44:201. doi: 10.1186/s13046-025-03456-x (PMC12247437; doi:10.1186/s13046-025-03456-x)
Supplement: Supplementary file 1 — Supplementary Material 1 [file 13046_2025_3456_MOESM1_ESM.docx]

**Supplementary Table 1** Sequences of primers used in qPCR.

| **Gene** | **Forward primer sequence (5′-3′)** | **Reverse primer sequence (5′-3′)** |
| --- | --- | --- |
| WDR3 (human) | GATGAAGCCCCTGAGGATCG | CAGCAGATTCCCTGAAGCCA |
| ANXA10 (human) | GTATGGCCGGGACCTGATTG | TGCAGCATGGTTTTGTGCTC |
| MYC (human) | GCAATGCGTTGCTGGGTTAT | TCCCTCCGTTCTTTTTCCCG |
| TIMM8A (human) | GGACTGTCATTCCGGACCTC | TTGGCAAGCAGAACACGTTG |
| WASF3 (human) | GACCGCGGACCGTTTTA | TGTTGTCGGGTCTAAGCTCT |
| GAPDH (human) | TGTGGGCATCAATGGATTTGG | ACACCATGTATTCCGGGTCAAT |

qPCR, quantitative real-time polymerase chain reaction.
