# Supplementary material for: WDR3 undergoes phase separation to mediate the therapeutic mechanism of Nilotinib against osteosarcoma
Source: J Exp Clin Cancer Res. 2025 Jul 11;44:201. doi: 10.1186/s13046-025-03456-x (PMC12247437; doi:10.1186/s13046-025-03456-x)
Supplement: Supplementary file 2 — Supplementary Material 2 [file 13046_2025_3456_MOESM2_ESM.docx]

**
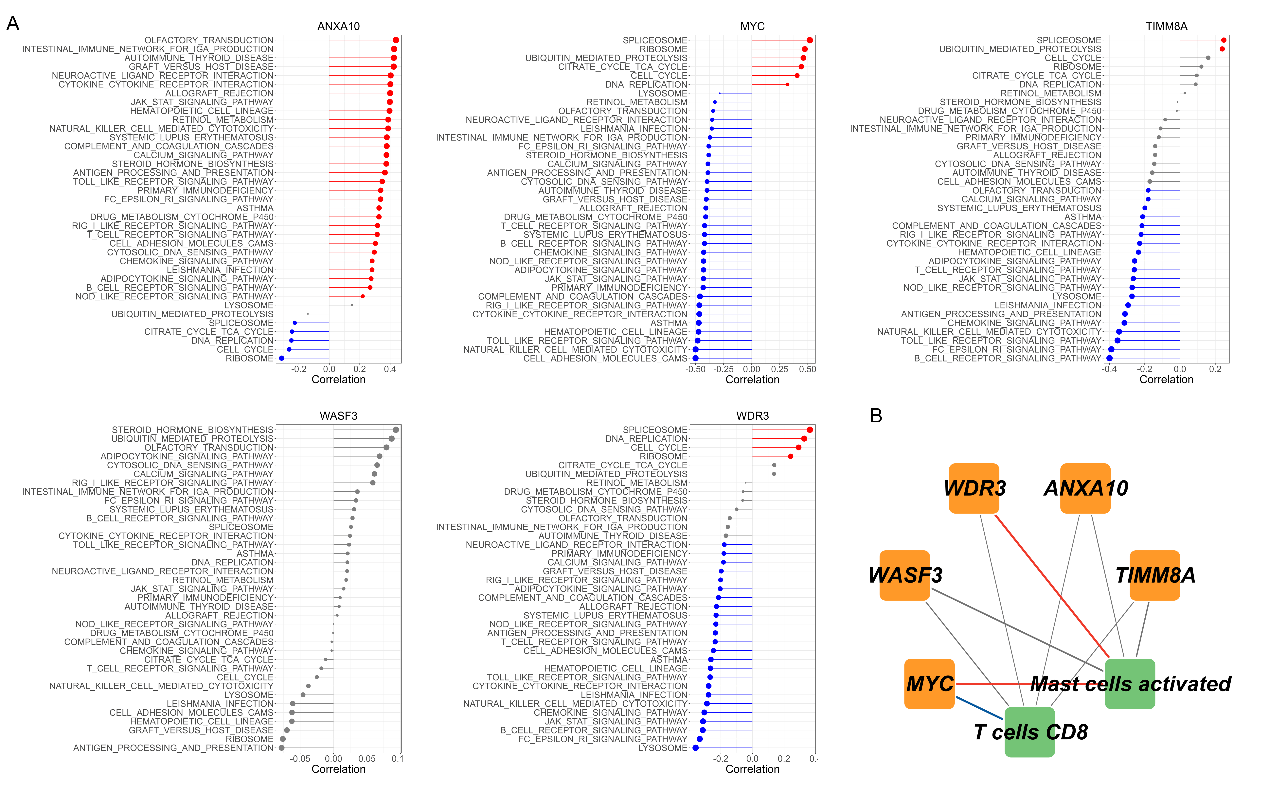
Supplementary Figure 1** Correlations between prognostic signatures with GSVA pathways (A) and immune infiltrating cells (B).
