# Supplementary figures and images for: WDR3 undergoes phase separation to mediate the therapeutic mechanism of Nilotinib against osteosarcoma
Source: J Exp Clin Cancer Res. 2025 Jul 11;44:201. doi: 10.1186/s13046-025-03456-x (PMC12247437; doi:10.1186/s13046-025-03456-x)

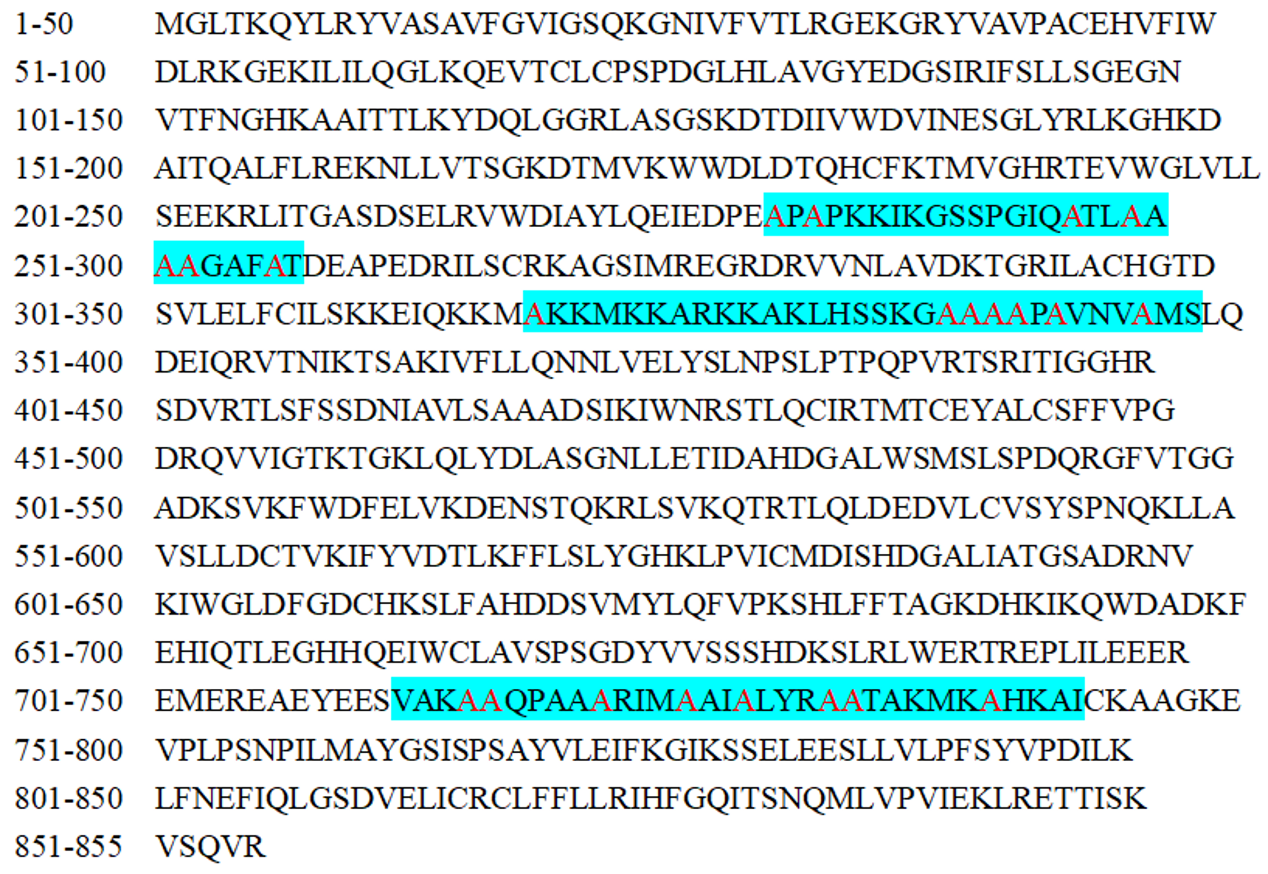
**Supplementary Figure 2** Amino acid mutation sites of three WDR3 IDR mutants.

Supplement: Supplementary file 3 — Supplementary Material 3 [file 13046_2025_3456_MOESM3_ESM.docx]
